# Mesoporous silica nanocarriers as drug delivery systems for anti-tubercular agents: a review

**DOI:** 10.1098/rsos.220013

**Published:** 2022-06-08

**Authors:** Josephine Oluwagbemisola Tella, Joseph Adeyemi Adekoya, Kolawole Oluseyi Ajanaku

**Affiliations:** Department of Chemistry, College of Science and Technology, Covenant University, Ota 112212, Nigeria

**Keywords:** tuberculosis, mesoporous silica nanoparticles, nanomedicine, drug delivery, anti-tubercular agents

## Abstract

The treatment and management of tuberculosis using conventional drug delivery systems remain challenging due to the setbacks involved. The lengthy and costly treatment regime and patients' non-compliance have led to drug-resistant tuberculosis, which is more difficult to treat. Also, anti-tubercular drugs currently used are poor water-soluble drugs with low bioavailability and poor therapeutic efficiency except at higher doses which causes drug-related toxicity. Novel drug delivery carrier systems such as mesoporous silica nanoparticles (MSNs) have been identified as nanomedicines capable of addressing the challenges mentioned due to their biocompatibility. The review discusses the sol–gel synthesis and chemistry of MSNs as porous drug nanocarriers, surface functionalization techniques and the influence of their physico-chemical properties on drug solubility, loading and release kinetics. It outlines the physico-chemical characteristics of MSNs encapsulated with anti-tubercular drugs.

## 1. Introduction

Tuberculosis is one of the world's deadliest infectious diseases caused by *Mycobacterium tuberculosis*. Around 10 million people across the globe were affected in 2020 by the disease [1]. Many efforts have been made towards completely eradicating the disease, but it comes with setbacks [2]. The treatment of drug-susceptible tuberculosis involves using a lengthy and complicated treatment regime that is costly and comes with adverse side effects and high toxicity. It has contributed to non-compliance to patients’ treatment regime, resulting in therapeutic failure and, consequently, the emergence of drug-resistant tuberculosis (multi- and extended drug-resistant tuberculosis), which is more costly and difficult to treat. In addition, several promising anti-tubercular drugs have been developed to treat and manage tuberculosis. Still, many of them are poorly water-soluble drugs with poor permeation and metabolic stability [3]. Thus, having low bioavailability and poor therapeutic efficiency [4]. To overcome the challenges encountered in treating and managing tuberculosis, more advanced drug delivery systems need to be designed and developed to enhance the therapeutic efficiency of anti-tubercular drugs.

Through the development of nanoparticle-based drug delivery systems, nanotechnology has created a platform for overcoming the challenges encountered in the treatment and management of diseases by seeking to improve drug bioavailability and decrease their side effects [5]. The main aim is to minimize drugs' concentration and dosage frequency, thereby creating a more effective and patient-compliant treatment regime with easy administration and improved safety [6,7]. It also has unique features which protect drugs from enzymatic degradation and metabolism to enhance the concentrations of drugs at their target sites. Consequently, the therapeutic efficiency of the medicines is improved [8,9]. Varieties of nanocarriers have been explored for developing nanoparticle-based drug delivery systems, one of which is porous materials.

Porous materials belong to a distinctive class of materials. They have porous structures of low-density solids with unique pore structures, composition and sizes, responsible for their different physico-chemical properties [10–12]. Some of these properties include large surface areas [13], high selectivity [14], low densities [15], excellent permeabilities [16] and low refractive coefficients [17]. Due to these properties, their popularity in technology-driven sectors has increased as their applications cut across energy conversion and storage, pharmaceuticals, medicine, transportation and catalysis [18,19].

Porous materials have different pore shapes such as hexagonal [20], spherical [21] and cylindrical [22]. They have internal [23] and external surfaces [24] responsible for their selective functionalization [25]. Their pore walls can interact with atoms, ions and molecules and have flexible spaces that allow the loading and release of solid particles, liquid and gaseous molecules in a controllable manner, giving them scientific and technological importance [26].

The porous structures of these materials can be made up of organic, inorganic and a combination of inorganic and organic composite materials. The organic porous materials are carbon-based materials, including carbon nanotubes such as nanofibres, graphite, single-walled and multiple-walled carbon nanotubes. Inorganic porous materials comprise metals, metal oxide-based materials and quantum dots (metalloid materials) such as Al, Si, Zn, Al_2_O_3_, Fe_2_O_3_, ZnO and ZnS. Inorganic and organic composite porous materials are also known as hybrid materials and are made up of organic–inorganic, organic–organic and inorganic–inorganic materials [27]. Inorganic nanoparticles have gained prominence as they possess better chemical stability, mechanical strength, microbial resistance and biocompatibility than organic nanoparticles [28].

According to the International Union of Pure and Applied Chemistry (IUPAC), porous materials can be classified into three categories based on the diameters of their pores, which are micropores (less than 2 nm), mesopore (2–50 nm) and macropore (greater than 50 nm), respectively [29–31]. The efficient and commonly used materials are the mesopores, as they possess properties that have made them ideal potential candidates for nano-catalysis, nano-fabrication and nanomedicine, especially drug delivery [32,33]. These properties include; large surface areas [34], modifiable pores [35], high porosities [36], mechanical stability [37] and good thermal stability [38].

The review gives a brief overview of mesoporous silica nanoparticles (MSNs) as drug delivery systems, focusing on their sol–gel synthesis, physico-chemical properties, surface functionalization, drug loading and release methods and a summary of the physico-chemical properties of MSNs encapsulated with anti-tubercular drugs.

## 2. Mesoporous silica nanoparticles

MSNs have an inorganic framework commonly synthesized by the reaction of sodium silicates or silica tetraethyl orthosilicate (inorganic silica source) with a surfactant micelle, usually quaternary ammonium salts [39]. Parameters such as the morphology of surfactants, a silica source, ionic strength, ageing time, temperature and pH conditions are vital for synthesizing porous silica materials. They influence the pore size and volume surface area structures MSNs [32].

MSNs came into the limelight in the 1990s when researchers from Mobil oil company synthesized a silica-based material MCM-41 (mobile crystalline material) from aluminosilicate gels using a liquid-crystal template mechanism in 1992 [40]. MCM-41 has a two-dimensional (2D) hexagonal pore structure [41]. It is prepared using cationic surfactants under basic conditions with a pH of 8.5–12 [42]. When the ratio of cationic surfactants to silica source is less than 1, the predominant pore shape of the mesoporous silica material synthesized is hexagonal [43,44]. MCM-48 and MCM-50 having three-dimensional (3D) cubic [45], and the lamellar-like [46] arrangements also were synthesized the same way as the MCM-41 mesopores using a varying cationic surfactant to silica source ratio. When the cationic surfactant to silica ratio was greater than 1, a cubic pore structure of MCM-48 was formed. Upon further increase, the formation of lamellar-like pore structures of MCM-50 occurred [43].

**Figure 1.**
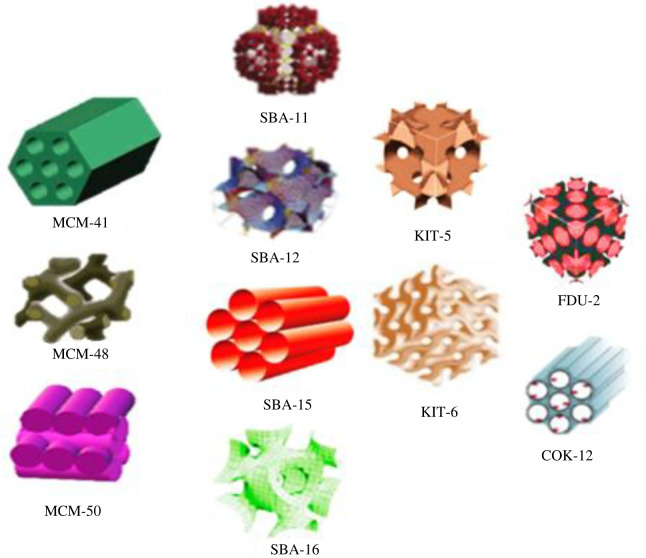
Types of mesoporous silica nanoparticles.

After the invention of MCM, other mesostructured materials such as SBA-11, SBA-12, SBA-15 and SBA-16 with cubic, 3D hexagonal, 2D hexagonal and cubic cage pore symmetry, respectively, were synthesized using non-ionic triblock copolymers such as poly(alkylene oxide) block copolymers and alkyl poly(ethylene oxide) (PEO) oligomeric surfactants as templates [47]. This group of highly ordered mesoporous structures was first synthesized by the University of California, Santa Barbara, and was named Santa Barbara amorphous (SBA) type material. Technische Universiteit Delft (TUD-1) was developed at Technical Delft University; COK-12 from the Center for Research Chemistry and Catalysis, KIT-5 from Korean Advanced Institute of Science and Technology and HMM-33 (Hiroshima mesoporous material-33) are also mesoporous materials synthesized with various pore sizes and symmetry [48,49]. The commonly used MSNs for drug delivery include MCM-41, MCM-50, SBA-15 and SBA-16 [50,51]. Figure 1 and table 1 represent some types of MSNs and a list of MSNs used for drug delivery with their characteristic properties, respectively.

**Table 1.** List of mesoporous silica materials used for drug delivery and their characteristic properties. MCM, mobile crystalline materials; SBA, Santa Barbara amorphous.

MSN family	MSN type	Pore symmetry	Pore size (nm)	Pore volume (cm^3^ g^−1^)	References
M41S	MCM-41	2D hexagonal P6 mm	1.5–8	>10	[41]
	MCM-48	3D cubic Ia3d	2–5	>10	[45]
	MCM-50	lamellar p2	2–5	>10	[46,52]
SBA	SBA-11	3D cubic Pm3 m	2.1–3.6	0.68	[53]
	SBA-12	3D hexagonal P6_3_/mmc	3.1	0.83	[52]
	SBA-15	2D hexagonal p6 mm	6–0	1.17	[54]
	SBA-16	cubic Im3 m	5–15	0.91	[55]

The use of mesoporous materials for drug delivery began in 2001 with the synthesis of a silica-based mesoporous material MCM-41 for the delivery of ibuprofen [56]. MSNs have unique properties which give them an edge over conventional drugs delivery systems, which includes:

(i) Controllable pore morphology and structures with large surface areas (700–1000 m^2^ g^−1^) and pore volumes (0.6–1 cm^3^ g^−1^) are essential for the loading and release of an extensive range of drug molecules [57,58].
(ii) Well-ordered pore structures (individual pore channels that do not interconnect) and modifiable pore sizes essential for modifying drug molecules’ loading and release kinetics [59].
(iii) Their low cytotoxicity and biocompatibility with cells [51,60] due to the ability of silica to quickly decompose into harmless silicic acid [61,62].
(iv) Host–guest interaction between silanol groups on silica surfaces of the host and an extensive range of functional groups from guest molecules; the silanol groups present on the two functional surfaces (cylindrical pore and exterior particle surfaces) of MSNs make selective functionalization possible, which enhances the adsorption and release of different drug molecules [63,64].
(v) Capable of forming metal ion complexes such as Mn–MCM 41 [65], Fe–Mn [66], Al–Mn [67] and Au MCM [68] through conjugation with metal ions. These metal complexes help to enhance the therapeutic profile in drug delivery and assist in diagnostics [69].
(vi) Surface areas of silica walls are hydrophilic and can trigger the fast dissolution of drug molecules by enhancing the wetting and dispersion of the drug molecules [70]. These surfaces can also be modified with hydrophobic functional groups for the easy adsorption of hydrophobic drugs [71]. The method can be used to prolong the release kinetics of some drug molecules by reducing the surface wettability of the mesoporous material [72].
(vii) The inorganic matrix protects drug molecules from enzymatic denaturation, temperature and pH variations, thereby conserving the chemical stability of the drug molecules [71].

### 2.1. Mesoporous silica nanocarriers: synthesis and chemistry

**Scheme 1.**
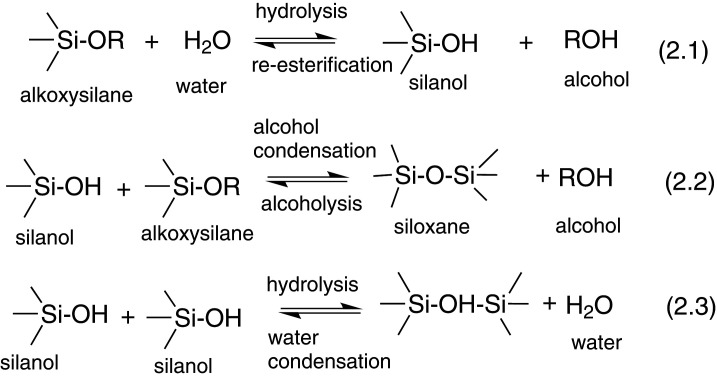
General reaction scheme for sol–gel synthesis.

Various methods can be used to synthesize mesoporous silica nanocarriers with various shapes and physico-chemical properties. The most common method used is sol–gel synthesis. It entails the formation of a colloidal solution (sol) from hydrolysis and condensation reactions of alkoxide monomers, which acts as a precursor for the formation of a distinct network (gel) of polymers or particles [73,74]. The synthesis of MSNs involves the hydrolysis and condensation of silanes (Si(OX)_4_). It occurs within an aqueous solution in an acidic or basic catalyst such as HCl or NH_3_ [75], which aids the reaction kinetics of the two processes. Where X is usually OEt or OMe or an organosilane ([(XO)_3_ Si]*_n_*-R and where R belongs to an organic group, *n* ≥ 1) [76]. Reactive silanolates species (=Si–O–) are formed during hydrolysis and are further condensed with other silanes or organosilanes to form covalent siloxane bonds (Si–O–Si) with increasingly bigger oligomers. Generally, the sol–gel process can be described using three reactions stated in scheme 1.

The first reaction illustrates the formation of silanol groups (Si–OH) through the hydrolysis of alkoxysilanes. The alkoxide group (OR) is replaced with a hydroxyl group (OH) (equation 2.1). The reaction rate is dependent on the water to alkoxide ratio, the pH value, the solvent and catalyst employed. The second reaction illustrates the condensation reaction of the silanol groups formed with an alkoxide or silanol group to create siloxane bonds (Si–O–Si) and by-products alcohol (ROH) (equation 2.2) or water (equation 2.3).

### 2.2. Main chemical constituents used in the synthesis of MSNs

**Scheme 2.**
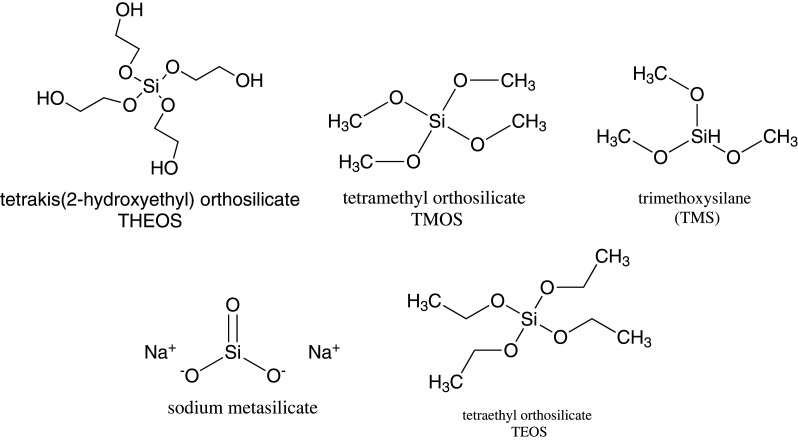
Chemical structures of some silica sources.

**Scheme 3.**
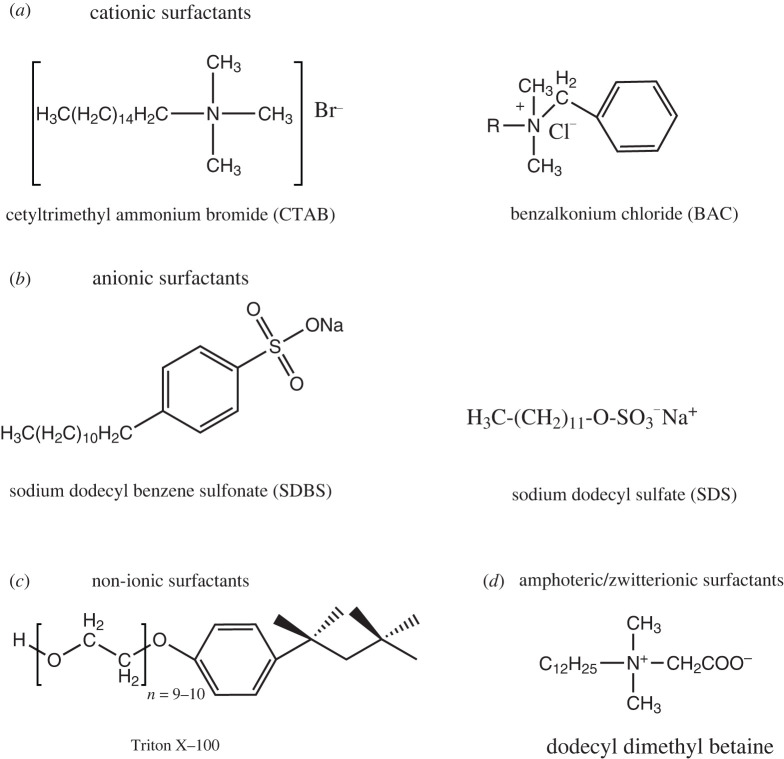
Chemical structure of surfactants.

The synthesis of MSNs requires three main components. These are inorganic silica source/precursor, a surfactant that serves as a structure directing agent/template and a catalyst. The examples of silica sources include tetramethyl orthosilicate (TMOS), tetraethyl orthosilicate (TEOS), tetra methoxy vinyl silane (TMVS), tetrakis (2-hydroxyethyl) orthosilicate (THEOS), tetra butoxy silane (TBOS), tetra propyl ortho-silicate (TPOS), trimethoxy silane (TMS) and sodium metasilicate (Na_2_SiO_3_) [77,78]. Schemes 2 and 3 show the chemical structures of some of these silica sources and surfactants, respectively.

The surfactants commonly used can be classified into the following:

(i) *Cationic surfactants*. These have a positively charged hydrophilic head (polar group) and a non-polar group (hydrophobic tail). The majority of these surfactants have alkali hydrophilic and methyl ammoniums such as cetyltrimethylammonium chloride (CTAC), cetyl trimethyl ammonium bromide (CTAB) and hexadecyltrimethylammonium (HDTMA) [79,80].
(ii) *Anionic surfactants*. These surfactants have a negatively charged hydrophilic head (polar head) and long hydrocarbon tail (non-polar end). They consist of sulfated (R-OSO_3_Na) and sulfonated (R-SO_3_Na) compounds [81].
(iii) *Non-ionic surfactants*. These are neutral surfactants that have a non-dissociable type of hydrophilic head, such as amide and phenol and cannot ionize in an aqueous solution. Examples include Triton X-100, polysorbate, Pluronic F127 and Pluronic P123 [82].
(iv) *Amphoteric/zwitterionic surfactants*. These surfactants have positive and negative charges on their hydrophilic ends, which cancel out each other, producing a zero net charge called zwitterionic. Examples include phospholipids, betaines or sulfobetaines and amino acids [83].

The type of catalyst used for the sol–gel synthesis of MSNs can be either acid or base is depending on the reaction (hydrolysis or condensation) that is faster than the other. Acid-catalysed reaction, which produces numerous small silica particles or a network of gels, is used when hydrolysis is faster than condensation reaction. By contrast, base-catalysed reaction responsible for forming larger silica particles or solid spheres is used when condensation is faster than hydrolysis reaction [44]. Diethanolamine (DEA), triethanolamine (TEA), hydrochloric acid, ammonia and sodium hydroxide are used commonly as catalysts [18,84–86].

### 2.3. Parameters considered in the synthesis of mesoporous silica nanoparticles

Various parameters such as surfactants, co-surfactants, a silica source, temperature and pH can directly or indirectly affect the morphology and textural properties of fabricated MSNs [76,87].

#### 2.3.1. Surfactants

Surfactants (surface active agents) have an essential role in synthesizing MSNs. They serve as templates for the growth of mesoporous materials, and the type of surfactants used determines the structures of the materials [44]. Surfactants capable of forming micelles above the critical micellar concentration (CMC) such as CTAB are commonly used by material scientists. When the surfactants have high concentrations above their CMC, the micelles change their shapes from spheres to cylinders and hexagonal channels. Larger pores can be created with the aid of a swelling agent. Mesoporous materials with well-defined pores can be synthesized by using a swelling agent and changing the surfactant's type and quality [43].

#### 2.3.2. Co-surfactants

Co-surfactants, majorly alcohols such as ethanol [88] and butanol [77], affect the pore size and ductility and affect the shapes of the pores as their concentration increases. As the concentration of co-surfactants increases, MSNs tend to lose their spherical shapes, and amorphous particles with various disordered pore sizes are formed [89,90]. Their ability to control the morphology and pore size of MSNs enhances the drug delivery potential of MSNs [82]. Surfactants used in synthesizing mesoporous silica materials can also play the role of co-surfactants.

#### 2.3.3. Solvents

Solvents also have essential roles to play in the synthesis of MSNs. The most efficient and typical examples of alcohol include ethanol, propanol, butanol and pentanol. Alcohols enhance pore formation and alter the sizes of mesopores. However, the morphology and shape of mesoporous materials are minimally affected by alcohols with low evaporation rates and high molecular weights [91]. The channel rotations of mesoporous materials are also modified by alcohols [92].

In addition, alcohols assist in the removal of surfactants after the synthesis of MSNs. Alcohol has been used as a solvent to aid the growth of cylindrical pores during the synthesis of radial MCM-48 [92]. Those with high boiling points have been used with solvents to remove surfactants, which prevented the agglomeration of the synthesized mesopores [93]. Furthermore, alcohol can also be used as a co-solvent [94]. Alcohols with long chains can be used to transit from one phase to another. For instance, after synthesizing a hexagonal phase in MCM-41, hexanol was used by Ågren *et al*. [95] to create a new lamellar phase.

#### 2.3.4. Silica sources

The synthesis of well-ordered MSNs requires precursors such as sodium silicates, colloidal solutions and organosilanes [96], such as TMOS, TEOS, TPOS and TMS [77]. TMS forms silicate mesoporous structures more rapidly than other precursors [97]. As the size of the alkoxy groups in silane increases, the rate of hydrolysis decreases due to steric hindrance (spatial effects), especially in highly branched silica sources [98].

#### 2.3.5. Temperature

The synthesis temperature is critical in determining MSNs' final properties as mesoporous materials can be synthesized between 10°C and 130°C, of which 25°C is regarded as the most appropriate [82]. Two essential factors to consider in terms of temperature are cloud point (CP) and critical micelle temperature (CMT). The CMT of surfactants should be lower than the temperature used during synthesis [97].

#### 2.3.6. pH

MSNs are either synthesized under acidic or alkaline conditions as neutral conditions do not favour the synthesis of well-ordered mesoporous structures due to high rates of polymerization and transverse bonding [99]. However, well-ordered mesoporous materials can be synthesized under neutral conditions by adjusting the hydrolysis and condensation of the silica precursors and using fluorine as catalysts [100]. The polymerization and creating adjustable silicate species networks occur under alkaline conditions with a pH of 9.5–12.5 using silica precursors such as TEOS, colloidal solutions and Na_2_SiO_3_ [79]. Under alkaline conditions, pH changes occur during synthesis. Silica hydrolyses at the beginning of the reaction; there is a decrease in pH and a little increase during the condensation of silica species [79]. Moreover, a similar trend can be observed under highly acidic conditions when the rate of mesoporous silica synthesis increases with a decrease in pH and the rate of silica precipitation increases in the presence of high concentrations of acid catalysts [82].

### 2.4. Surfactant removal after synthesis

To use the synthesized MSNs for their various drug applications, the complete removal of surfactants from nanoparticles is essential for three main reasons:

(i) *Cytotoxicity*. Most surfactants used to synthesize MSNs are toxic to living cells. Some can cause cell death at high concentrations by interacting with the phospholipids present in cell membranes. Therefore, removing these pore-forming agents is necessary before their use for biological applications [101].
(ii) *Pore accessibility*. Surfactants tend to reduce the pore volume of synthesized MSNs. The pore volume affects the drug loading and release rates of mesoporous materials. Large pore volumes can reduce the tendency of intense drug–drug interactions, which aids intermolecular interactions between drug molecules and pore walls, resulting in high loading capacity [102]. The presence of surfactants in pore walls reduces the small pore volumes and minimizes drug molecules' loading and release rates.
(iii) *Surface modification*. Surfactant removal from pores enhances the surface modification of the synthesized nanoparticles as it makes the pores more accessible to an extensive range of functional groups such as amino acids, thiol, small organic phosphates, carboxyl groups and phospholipids [62]. The two silica surfaces (cylindrical pore and exterior particle surfaces) can be functionalized with the same functional groups or with two different functional groups using two basic synthetic strategies; co-condensation or post-synthetic grafting [103].

Surfactants can be removed after the formation of mesoporous silica structures using the following methods:

#### 2.4.1. Calcination

Calcination involves subjecting the synthesized MSNs to temperatures as high as 800°C for the decomposition of the surfactant used. Hollow cylinders of inorganic materials are formed in the process [32]. This method comes with disadvantages such as surface modification and high temperature and energy requirements. The Si–OH bonds present on the surface of the synthesized mesoporous silica material is converted to Si–O–Si bonds at high temperatures, resulting in compression of the pores and the surfaces. As a result, the pore size is affected, and the particle becomes hydrophobic [44]. In addition, calcination causes dehydration and cross-linking between particles, causing irreversible aggregation of particles, challenging to redisperse back into solitary particles [93,104].

#### 2.4.2. Solvent extraction

Solvent extraction is a mild alternative for calcination, requiring high thermal treatment. Based on the type of surfactant and the experimental conditions, either acidic or basic, solvents can be used to extract the synthesized nanoparticles. Examples of solvents used include ammonium nitrate [105], water [106], ethanol [107], hydrochloric acids [108] and other alcohols [109]. Compared with calcination, solvent extraction has less impact on the porosity and structures of synthesized mesoporous materials. However, in most cases, complete surfactant removal cannot be achieved with solvent extraction, but it is possible to re-use recovered surfactants. This method is ideal when complete removal of surfactants is not required [110].

#### 2.4.3. Chemical-assisted oxidation

Hydrogen peroxide is commonly used as a chemical oxidant to remove surfactants through oxidation reactions [111]. This method increases pore diameters and reduces total pore volume and surface areas [112]. Also, it increases the number of silanol groups present on the silica walls compared with calcinated samples [113]. An acid such as HNO_3_ is frequently added with hydrogen peroxide for surfactant removal [112]. Other chemical oxidants that have been used include; ozone [114], KMnO_4_–H_2_O_2_ and NH_4_ClO_4_ [115,116].

#### 2.4.4. Microwave digestion

Microwave digestion is the fastest way to remove surfactants from mesopores. It involves exposing the synthesized mesoporous materials to microwave radiation for about 2 min after being suspended in an HNO_3_–H_2_O_2_ solution [117] or hexane and ethanol [118]. This method does not affect the textual properties of synthesized mesoporous materials. It increases the concentration of silanol groups compared with calcinated samples and yields higher pore volume, size and larger surface areas [117].

### 2.5. Surface functionalization

**Scheme 4.**
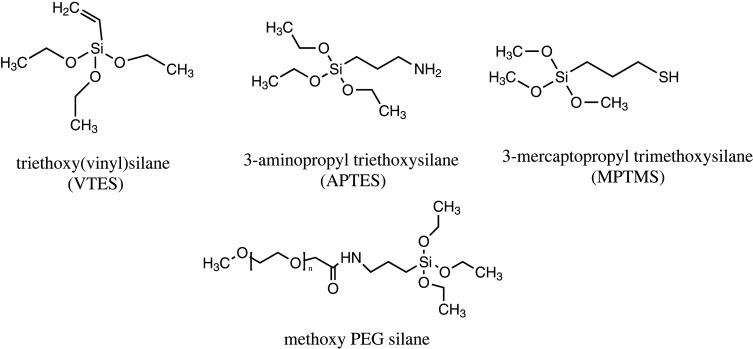
Chemical structure of commonly used organosilanes.

The physical and chemical properties of MSNs can be enhanced through surface functionalization with different functional groups for improved drug adsorption, delivery and sustained release at target sites [119]. The presence of silanol groups on the surface of silica walls makes functionalization with different functional groups easy through covalent grafting with organic silanes ((RO)_3_SiR^1^) [120]. Commonly used organic silanes include vinyltriethoxy silane (VTES), 3-aminopropyl triethoxysilane (APTES), methoxy-PEG-silane and 3-mercaptopropyl trimethoxysilane. It makes MSNs versatile and suitable to perform specific tasks. Scheme 4 provides the chemical structures of commonly used organic silanes. Examples of functional groups surfaces that are modified include; carboxyl groups (COOH), amino-containing polymers such as polyethylenimine (PEI), phospholipids, polyethylene glycol (PEG), small organic phosphates and thiols [62]. Functional groups such as 3-aminopropyltriethoxysilane [121], polylysines [122] and polyethylenimine [123] are used to modify negatively charged surfaces, such as carboxylic acids [124]. Hydrophobicity can also be reduced using diethoxydimethyl silane [125], trimethylchlorosilane [126] and polymethyl hydrosiloxane [127] which helps to enhance the drug loading capacity of hydrophobic drugs [72]. There are three significant functionalization sites on MSNs: pore entrances, pore walls and interior/exterior surfaces of the nanoparticles [128]. Drug molecules are encapsulated to these sites by covalent bonding, hydrogen bonding, van der Waal interactions or electrostatic binding based on the functional groups attached to the sites [129]. MSNs can be chemically modified using two main methods: post synthesis or grafting and co-condensation method [130].

#### 2.5.1. Post synthesis or grafting method

Post synthesis entails grafting organo-trialkoxysilanes or organo-trichlorosilane with synthesized MSNs after surfactant removal by calcination or extraction. The functional groups attached to MSNs are located at the exterior surface or the openings of the mesopores. One of the significant challenges with this method is the possibility of having the mesopore openings blocked with functional groups, which causes the heterogeneous or non-uniform distribution of functional groups on the silica matrix [131,132]. However, this method is suitable for the functionalization of exterior surfaces of mesopore walls [133].

#### 2.5.2. Co-condensation method

**Figure 2.**
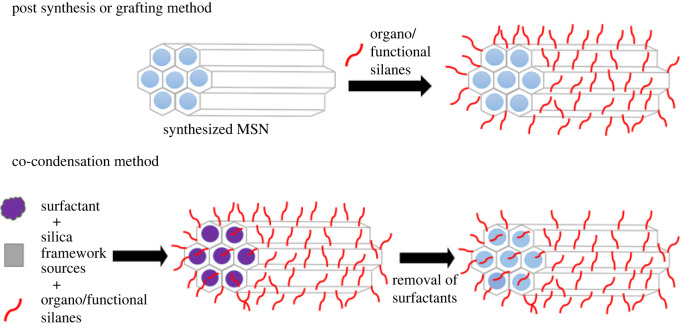
Schematic representation of surface functionalization methods.

Co-condensation involves the synthesis and functionalization of MSNs in a one-pot route synthesis. During mesoporous synthesis, organo-alkoxy silanes are introduced together with the silica source and surfactant at the condensation stage [103]. Surfactant is removed using ion-exchange with either an ethanolic solution of hydrochloric acid [108] or ammonium nitrate [105]. This method allows functional groups to be grafted to the outer and inner surfaces of the silica walls, as illustrated in figure 2 [134].

### 2.6. Effect of synthesis parameters on the physico-chemical properties of mesoporous silica materials

The effects of synthesis parameters on the physico-chemical properties of mesoporous silica materials are discussed as follows:

#### 2.6.1. Pore size and shape

The pore size and shape of mesopores determines the type and amount of drug molecules that can be encapsulated within the MSNs and also the dissolution rates of drugs [135]. The appropriate pore size has to be used to prevent the premature release of drug molecules [136,137]. With the help of the mesoscale channels present in MSNs, drugs can be preserved in a non-crystalline state [138]. The type, chain length and concentration of surfactants used as templates can control the final pore size [135,139]. Jana *et al*. [140] investigated the effect of various alkyl chain lengths of tetra-alkylammonium salts (commonly used surfactants) on the pore sizes of MSNs. It was observed that an increase in the surfactant chain length from C8 to C22 can increase the pore size from 1.6 to 4.2 nm. Relevant studies have also shown that the pore size can be up to 4.1 nm by adjusting the surfactant chain length [141,142].

Also, experimental conditions such as reaction time and temperature, the choice of silica precursor and catalyst concentration have essential roles in determining the pore sizes of MSNs [143]. The pore shapes also affect the drug loading and release rates of mesoporous materials, either 2D pore or 3D interconnected structures [144].

Moreover, effective loading of drug molecules requires using the appropriate matrix as mesopore diameters determine the size of drug molecules confined within the matrix [145]. Drug molecules that are smaller than the diameters of the mesopore's cavities are absorbed within the inner surface of the mesopore. By contrast, molecules larger than the diameters of the mesopore's cavities are absorbed on the external surfaces of the cavities. Consequently, pore diameter serves as a size-selective adsorption factor [63]. To achieve an adequate drug loading capacity, the ratio of the pore diameter to the size of the drug must be significantly greater than 1. The drug loading rate improves as the ratio increases due to increased diffusion [137].

Pore diameter also plays a vital role in drug release kinetics as they serve as a drug release rate modulator. The influence of pore size was observed when different MCM-41 matrices were synthesized using cationic surfactants having different chain lengths for the delivery of ibuprofen. A decrease in the pore diameter caused a decrease in the release rate of ibuprofen [136]. As a result, mesopore diameters can be adjusted to regulate the release kinetics of drugs [63]. Likewise, pore volume is a crucial factor to consider when high amounts of drug molecules and large drug molecules such as proteins are to be absorbed as they influence the drug loading properties of nanocarriers. Large pore volumes can prevent drug–drug interactions, which aids drug–pore wall intermolecular interactions resulting in high loading of mesopore channels [102,146].

Meanwhile, drug loading is considered a surface-related phenomenon of which the total surface area is a crucial factor that influences it [147]. Total surface area refers to the sum of nanocarriers' inner and outer surface areas. It can be altered by the choice, type and concentration of surfactants used and surface functionalization. Typically, mesoporous materials have pore sizes of less than 15 nm, a total pore volume of 1–2 cm^3^ g^−1^ and a surface area of 1000 m^2^ g^−1^ [147,148]. Also, the specific surface area of matrices regulates the number of drug molecules retained within the matrices. As the specific surface area increases, there is more room for host–guest interactions, allowing a higher amount of drug molecules to be retained with a slower release rate. It was observed in alendronate loaded into SBA-15 and MCM-41 matrices. Alendronate released from SBA-15 (719 m^−2^ g^−1^) exhibited zero-order kinetics, while alendronate released from MCM-41 (1157 m^−2^ g^−1^) showed first-order kinetics [149].

#### 2.6.2. Particle morphology and surface charge

The particle size, shape and surface charge are essential factors determining mesoporous materials’ *in vitro* and *in vivo* drug delivery. MSNs with a diameter of less than 1 µm are highly sought in drug delivery as they have fast mass transport and excellent dispersibility compared with their bulk counterparts [87,150,151]. The surface charge and topology of MSNs affects their pharmacokinetics and accumulation at their target sites [152]. The cellular uptake of MSNs, their cellular interactions, distribution and elimination, are controlled by the particle size of the nanocarriers [153]. The surface charge also affects the cellular uptake and the *in vivo* immune response to mesoporous materials [152,154].

The particle size of MSNs is a factor that can be altered by specific parameters such as pH [155], reaction temperature [156], stirring rates [157], types of silica precursor [158] and additives such as functional organosilanes [159], TEA as a base alternative [160], co-surfactants [143] and gelatine [138]. The hydrolysis rates and condensation of silane bonds are greatly affected by pH, which subsequently controls the particle size of MSNs. Chiang *et al*. and Wu *et al*. reported an increase in the hydrolysis rate of TEOS as pH increased with large particle size. Also, an increase in the hydrolysis rates and polymerization of silica precursors as reaction temperature increased resulted in MSNs having larger particle sizes [87,150,156,161,162].

**Figure 3.**
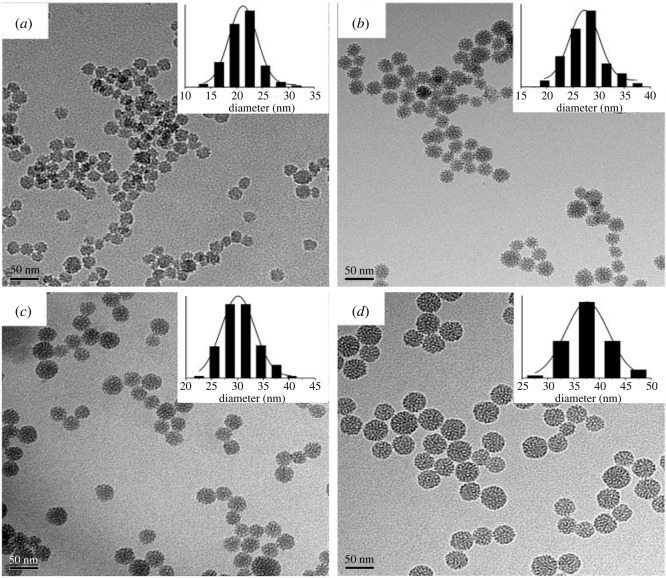
TEM images of MSNs synthesized with 0.06 g of TEA, stirring rate of 700 r.p.m. and different temperatures (*a*) 40°C (*b*) 60°C (*c*) 80°C and (*d*) 95°C (adapted from Lv *et al*. [163]).

**Figure 4.**
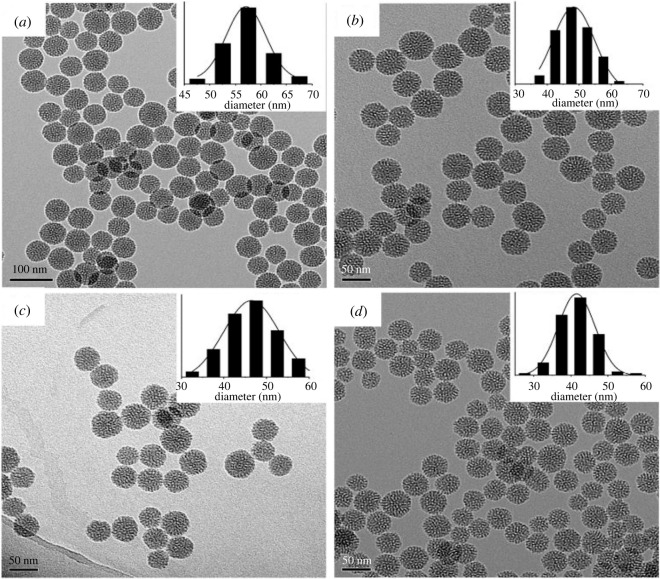
TEM images of MSNs synthesized with a stirring rate 400 r.p.m., reaction temperature of 95°C and different TEA concentrations (*a*) 0.06 g (*b*) 0.06 g (*c*) 0.12 g (*d*) 0.20 g (adapted from Lv *et al*. [163]).

**Figure 5.**
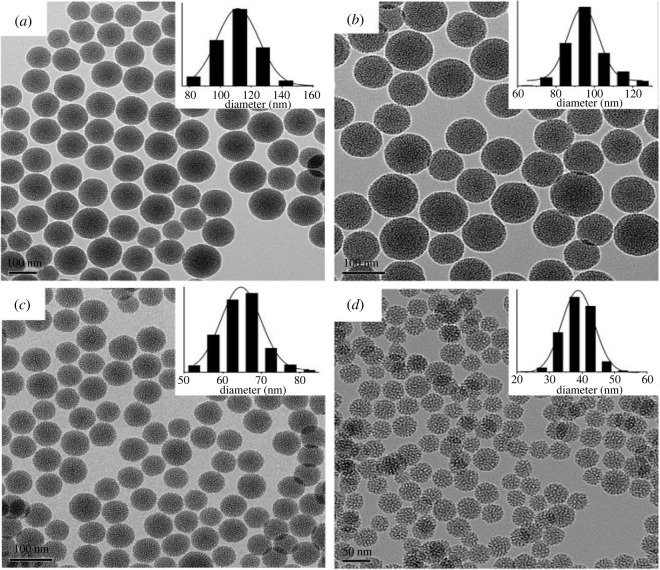
TEM images of MSNs sample synthesized with 0.06 g of TEA, 95°C reaction temperature and different stirring rates of (*a*) 100 r.p.m. (*b*) 200 r.p.m. (*c*) 300 r.p.m. (*d*) 10 000 r.p.m. (adapted from Lv *et al*. [163]).

The effect of base (TEA) concentration, reaction temperature and stirring rate on particle size was studied by Lv *et al*. [163]. An increase in temperature of 55°C resulted in particle size growth from 21 to 38 nm. At the same time, a decrease in particle size occurred for a slight increment in the base concentration. There was a decrease from 51 to 41 nm by adding 0.18 g of TEA. Also, increasing the stirring rate from 100 to 700 r.p.m. drastically reduced the particle size from 110 to 38 nm, with no further decrease occurring after the rate increased from 700 to 1000 r.p.m. The transmission electron microscopy (TEM) images shown in figures 3–5 represent the effects of stirring rate, base concentration and reaction temperature on the particle size of MSNs.

Moreover, various shapes of MSNs can be synthesized by modifying reaction conditions such as the temperature of synthesis, types of co-surfactants, stirring rate, additives and the molar concentration of silica source, surfactant, catalyst and water [52,135,164]. Slight changes in the molar ratios of reaction mixtures compositions and their acidity have been reported to affect the particle morphology of MSNs [165]. Shapes like spherical and silica rods were generated by Cai *et al*. [166] through molar concentration modification of CTAB, TEOS and NaOH/NH_4_OH. Discoid and spherical shaped MSNs were also synthesized by Naik *et al*. The pH of the reaction mixture was lowered, which reduced the condensation rate of silica and consequently lowered the local curvature energy resulting in the generation of discoid and spherical-shaped MSNs [167].

Various shapes of MSNs ranging from sphere to yolk shell-like structures with well-ordered size, shell structure, porosity and internal space were generated by Han *et al*. [168] through the adjustment of temperature of synthesis and quantity of dodecanol used as a soft template. Rod-shaped MSNs were synthesized by regulating the molar concentrations of reactants, the temperature of synthesis, addition of co-solvents such as heptane and increasing the amount of catalyst used [153,169,170].

In addition, using organo-substituted trialkoxysilanes as co-structure directing agents can also control the particle size and morphology of MSNs. Various shapes of MSNs with controlled particle size were fabricated using several alkyl-substituted silanes with different functionalities [171]. Similar results were reported by Sadasivan *et al*. using three different functional groups and an anionic surfactant route [172,173]. Specific shapes of MSNs can be generated by making use of dual-surfactant systems such as sodium dodecyl sulfate/HDTMA bromide [174], CTAB/dodecanethiol [168], CTAB/polystyrene-b-poly(acrylic acid) [89], CTAB Triton X-100 [175], CTAB/perfluorooctanoic acid [127] and CTAB/sodium dodecylbenzene sulfonate [176].

However, MSNs have a surface charge on both their outer and inner surfaces. Surface charges do not only affect nanoparticles' stability, opsonization, cellular interactions and bio-distribution [177]. The inner surface charge is known to influence the drug loading capacity of MSNs [178]. In addition, the anti-bacterial efficacy of nanoparticles is greatly influenced by surface charge. Nanoparticles with many cationic groups on their surfaces tend to neutralize the negative charge of the bacterial cell membrane, which results in contact death by inhibition of bacterial respiratory function or sterilization due to change in the number of surface charges resulting in bacterial dissolution [179].

### 2.7. Comparing the performance of MSNs with conventional drug dosage forms such as compressed tablets, 3D-printed drugs and polymeric nanoparticles

Compressed tablets are pharmaceutical dosage forms made by subjecting a dry granular powder to sufficient pressure to make the particles cohesive yet with the ability of the content of the tablets to be released predictably and reproducibly. Conventional drug dosage formation methods include direct compression, which involves multiple processes such as blending, mixing, milling and finally compression into tablets. The conventional production techniques are intended to be a large-scale mass production with a one-dose-fit-all approach that may not necessarily consider a patient's individual needs. The significant disadvantages of the traditional manufacturing process include being time-consuming and costly while also requiring highly skilled technicians. More recently, 3D printing technology is revolutionizing drug manufacturing in the pharmaceutical industry because it can curtail drug production from days to a few hours. When the production process is accelerated, it can lead to a more rapid release of the drug product into the market [180]. The ability to rapidly produce drug dosage by the 3D method brings about a substantial reduction in the cost of production, which is favourable to the economy of the process [181].

3D printing or additive manufacturing (AM) is used to transform a 3D digital model into a 3D physical subject by successive material deposition in a layer-by-layer mode. Among 3D printing strategies for drug production are stereolithography (SLA), binder jetting (BJ), powder bed printing (PBP), semi-solid extrusion (SSE) and inkjet printing (IP). The printed drugs can be generated by different processes that replace the ink with a desirable drug formulation and then released to a suitable substrate in an additive process. The substrate could be an edible sheet with a functionalized structure of specific hydrophobicity/hydrophilicity, porosity and permeability. The first step to manufacturing a 3D object involves designing a digital model of the desired 3D product by a unique CAD (the software comes in many forms and licences). Subsequently, the digital design is exported to a readable format for a system, mainly a stereolithography (SLA) file. Later, a slicer (3D printing software) converts the SLA file into a series of thin layers with an instruction tailored to generate the 3D object [182]. One of the most innovative aspects of AM is the ability to develop oral dosage forms with elaborate shapes and complex structures (like floating systems) which were previously impossible to produce or required laborious and cost-ineffective procedures. Furthermore, dosage forms with more-sophisticated shapes and geometries can be easily manufactured via a broad spectrum of AM techniques, including:

(i) dosage forms with an internal channelled honeycomb network or gyroid microstructure, where dimension adjustments could tailor drug release;
(ii) torus-shaped formulations achieving active pharmaceutical ingredients (API) zero-order release via fusion deposition modelling (FDM), SLA or PBP printing; and
(iii) composite multi-layered or shell–core formulations that could deliver one or more APIs at different rates, depending on their specific structure and sequence of layers.

FDM is usually introduced to design and produce a bilayer tablet consisting of rifampicin (RFC) and isoniazid (INZ) for the treatment of tuberculosis. Ghanizadeh Tabriz *et al*. [181] formulated INZ in hydroxypropyl cellulose (HPC) matrix to allow drug release in the stomach (acidic conditions), and RFC was formulated in hypromellose acetate succinate (HPMC-AS) matrix to afford drug release in the upper intestine (alkaline conditions). This design could offer a better clinical efficacy by minimizing the degradation of RFC in the acidic condition and potentially avoiding drug–drug interaction. The bilayer tablet was prepared by fabricating drug-containing filaments using hot-melt extrusion (HME) coupled with 3D printing [181,183].

The fabrication steps involving 3D printing are clean, and the material wastes are negligible, making initially thrashed raw materials to be explored while also increasing compliance and accessibility of drugs [184]. As a result, there is an increase in research into 3D pharmaceutical printing techniques because of the reduced cost-benefit. However, there is a need to consider the potential product liability implications. Based on its role in providing the product blueprint alone, the firm may be partially responsible if an adverse incident or product defect claim arises. Another limitation of this approach is cyber risk. The proliferation of counterfeit medicines is perhaps the industry's most significant concern with 3D printing. Printers are much more vulnerable to hackers than traditional manufacturing processes, and the short production time magnifies the risk of counterfeits. In addition, the main challenge of the 3D printing technique is to convert the starting materials (drug and excipients) into ‘curable ink’ or a printable material [185].

Moreover, the safety and efficacy of 3D printers are often subjects of concern because traditional mass-manufacturing facilities are subject to intense oversight from regulatory bodies, which keeps products safer and provides solace to the insurers who cover them. However, the Food and Drug Administration cannot regulate every instance of 3D printing, so determining the safety of products developed and responsibility for adverse events is murky. Hence, there is a need to consider a more eco-friendly, safe, efficient and easily regulated approach domiciled within the purview of the pharmaceutical industry.

In this regard, research is leveraging multifunctional and stimuli-responsive mesoporous nanocarriers drug delivery systems that provide multiple benefits to overcome limitations of the traditional drug dosage forms, such as protection of the drug and enhanced bioavailability and targeted delivery to the disease site. Nanocarriers have exhibited tremendous successes in the targeted delivery of therapeutics to the desired tissues and cells with improved bioavailability, high drug loading capacity, enhanced intracellular delivery and better therapeutic effect [186].

Cheng *et al*. designed and synthesized a pH-responsive multifunctional MSN system comprising polydopamine, poly(ethylene glycol) and folic acid for targeted delivery of doxorubicin to bridge the limitations of drug delivery with the conventional drug fabrication modes. The authors observed the release of the encapsulated drug from this MSN–PDA–PEG–FA nanosystem in acidic pH and high anti-tumour activity [187]. Elsewhere multifunctional MSNs were prepared by decorating their surface with cationic polymers to facilitate electrostatic attachment and delivery of anionic therapeutics such as nucleic acids and siRNA [188]. In the recent decade, Yang *et al*. [189] developed disulfide-bridged degradable dendritic mesoporous organosilica nanoparticles (DDMONs) for therapeutic protein delivery to cancer cells. This DDMONs system showed a higher rate of glutathione (GSH)-responsive degradation and release of the therapeutic protein in B16F0 cancer cells. By contrast, the degradation of the nanoparticle was low in the normal HEK293t cells. Specifically, consideration must be given to exploring the great potentials of multifunctional MSN to deliver anti-tubercular drug candidates to ameliorate the problem of drug resistance and patient non-compliance with dosage routine owing to the dangerous endogenic side effects associated with the treatment regime. Therefore, a different treatment of the application of MSN in the anti-tubercular drug must be on the front burner.

### 2.8. Application of mesoporous silica nanoparticles for anti-tubercular drug delivery

Most existing and novel drugs are poorly water-soluble drugs with low absorption rates and poor bioavailability [190,191]. Poor drug solubility is due to the drugs’ high crystallinity, melting temperature and partition coefficient [192]. Solubility has a vital role in determining the efficiency of drugs as it affects their overall therapeutic potential. Due to their poor solubility, poorly water-soluble drugs are quickly expelled from the gastrointestinal tract before fully dissolved and absorbed into the bloodstream for circulation [193]. It leads to poor bioavailability and low dose proportionality, which in most cases, dose augmentation is required to achieve the right therapeutic blood concentration. Dose augmentation sometimes comes with topical toxicity within the gastrointestinal tract resulting in poor patient compliance as seen in the treatment of tuberculosis.

**Scheme 5.**
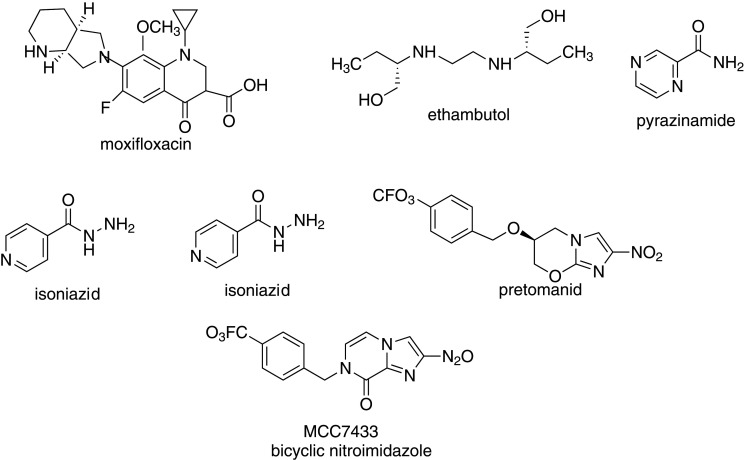
Chemical structures of some anti-tubercular agents encapsulated in MSNs.

MSNs have special features making them an excellent drug carrier that enhances the solubility of poorly water-soluble drugs [194]. Their bioavailability [195] makes them suitable for delivering anti-tubercular drugs. The spatial confinement of poorly soluble drug molecules encapsulated within the mesopores decreases the lattice energy and crystallization of amorphous drugs [196], which, as a result, increases their bioavailability and dissolution rates compared with crystalline drugs [195,197]. Also, dispersion and wetting of the encapsulated drugs are made possible by the hydrophilic large surface areas of MSNs, which enhances the dissolution rate of the drug molecules [70] and their drug loading capacities [198]. Table 2 summarizes the physico-chemical properties of anti-tubercular agents encapsulated in different types of MSNs, while scheme 5 provides the chemical structures of some anti-tubercular agents that have been encapsulated in MSNs.

**Table 2.** Physico-chemical characteristics of mesoporous silica nanoparticles encapsulated with anti-tubercular drugs. SSA, specific surface area; Vp, total pore volume; LC, loading capacity; EE, encapsulation efficiency.

anti-tubercular agent	MCM/morphology	silica source	structure directing agent	catalyst	synthesis method	surface modification	surfactant extraction process	encapsulation method	SSA *S*_BET_ (m^2^ g^−1^)	*V*_p_ (cm^3^ g^−1^)	pore size (nm)	particle size (nm)	LC (% w/w)	EE (%)	reference
pretomanid	MCM-41								503.4	1.053	2.04	250 ± 2.68	9		[199]
	(spherical)							rotary						≥86	
	MCM-41-NH_2_	TEOS	CTAB	NaOH	sol–gel	post synthetic	calcination	evaporation	251.84	0.539	1.89	268 ± 9.66	9.4		
	(honeycomb)					grafting									
	MCM-41-PO_3_^−^								477.34	1.011	1.86	208 ± 4.65	8.6		
	(honeycomb)														
MCC7433	MCM-41								448.81	0.945	—	185 ± 3.29			
	(spherical)							rotary						≥86	[199]
	MCM-41-NH_2_	TEOS	CTAB	NaOH	sol–gel	post synthetic	calcination	evaporation	—	—	—	258 ± 13.7	13		
	(honeycomb)					grafting									
	MCM-41-PO_3_^−^								—	—	—	176 ± 2.60			
	(honeycomb)														
moxifloxacin	AMS-6	TEOS	APES	—	sol–gel	—	calcination	rotary	534	0.65	—	300	40.4	—	[200]
	(spherical)							evaporation							
PA-824	AMS-6	TEOS	APES	—	sol–gel	—	calcination	rotary	513	0.55	—	300	28	—	[200]
	(spherical)							evaporation							
	MSNP-40						solvent					40	38.3	26.8	[201]
	(spherical)	TEOS	CTAB	Lysine	sol–gel	—	extraction	passive diffusion	—	—	9				
	MSNP-100											100	41.1	22.5	
	(spherical)														
	MSM	TEOS	CTAB	DEA	sol–gel	—	solvent	passive diffusion	816	1.0679	2.4	218 ± 46	—	52	[198]
rifampicin	(spherical)						extraction								
	MSN40e						solvent		483	1.3	11.7	47. ± 7.0	28.9	—	[202]
	(HMM)						extraction								
	MSN40c	TEOS	CTAB	Lysine	sol–gel	Co-condensation									
	(HMM)						calcination	centrifugation	483	1.3	11.7	47 ± 7.0	33.6	—	
	MSN80c														
	(HMM)						calcination		460	0.81	7.46	84.1 ± 17.4	38.2	—	
isoniazid								impregnation							[203]
pyrazinamide	MSN						solvent		875.8	1.029	3.86	90–100			
pyrazinoic acid	(honeycomb)	TEOS	CTAB	NaOH	sol–gel	—	extraction	rotary					—	—	
ethambutol								evaporation

The use of spherical and honeycomb-shaped MSNs for the encapsulation of poor water-soluble bicyclic nitroimidazole compounds, pretomanid and MCC7433 was studied by Ang *et al*. [199]. The surfaces of these materials were modified with organosilane surface modifiers APTES for amine groups and 3-(trihydroxysilyl)propyl methylphosphonate (THMP) for phosphonate group using post-synthesis grafting method. The nanoparticles were synthesized using the sol–gel method and encapsulated using the rotary evaporation technique. It was observed that the saturated water solubility of the encapsulated anti-tubercular compounds improved compared with the non-encapsulated compounds. Also, a high drug loading capacity of MCM-41 was reported, with MCC7433 having a slightly higher loading capacity than pretomanid. Furthermore, the encapsulated pretomanid and MCC7433 were observed to be an amorphous form compared with their crystalline non-encapsulated form [199]. Xia *et al*. encapsulated moxifloxacin (an 8-methoxy quinolone) and PA-824 (a nitroimidazole) in spherical shaped MSNs using the solvent extraction method. It was observed that moxifloxacin had a higher dissolution rate for both the encapsulated and non-encapsulated drugs in phosphate-buffered saline (PBS) buffer at a pH of 7.4. Non-encapsulated PA-824 showed a lower solubility with a poor release rate in PBS buffer due to its poor aqueous solubility. However, a faster dissolution rate of encapsulated PA-824 was reported than non-encapsulated PA-824. One-hundred percentage of the PA-824 loaded was released within 4 h, while non-encapsulated PA-824 had a 63% release rate after 4 h and an 80% release rate after 24 h. A 20% increase in the solubility of encapsulated PA-824 was attained compared with that of non-encapsulated PA-824 in the crystalline state. Furthermore, the X-ray diffraction patterns obtained showed no recrystallization of encapsulated moxifloxacin and PA-824, which were in an amorphous state compared with their highly crystalline non-encapsulated forms [200].

Mohseni *et al*. successfully synthesized spherical shaped MSNs by the sol–gel method and passive diffusion for encapsulation. The entrapment efficiency was observed to depend on the polarity index of the solvent used. Due to its low polarity index, dimethyl sulfoxide had a low entrapment efficiency, while water with a high polarity index also yielded low efficiency. However, methanol produced the highest entrapment efficiency of the three solvents used. They also studied the effect of temperature on the drug loading procedure employed. The entrapment efficiencies of 25% and 51% were reported at 4°C and 25°C, respectively, while RFC's fast degradation occurred at 45°C. The *in vitro* release rate of 60% was reported for the first 4 h, and 95% of RFC was released after 24 h, creating a biphasic release system of high and slow-release rate [198].

Furthermore, Subramaniam *et al*. synthesized Hiroshima type MSNs with particle sizes of 40 and 100 nm and encapsulated RFC in them using the passive diffusion method. It was observed that MSNs with the particle size of 40 nm (MSNP-Rif 40) had a lower loading capacity, 38.3%, than that of 100 nm (MSNP-Rif 100) 41.1%. The entrapment efficiency of 26.8% was reported for MSNP-Rif 40 and 22.5% for MSNP-Rif 40. Subramaniam *et al*. also studied the *in vitro* release rate of RFC from PBS buffer with a pH of 7.4 and acetate buffer with a pH of 5. It was observed that the smaller MSN particle size, MSNP-Rif 40, released about 10% of RFC in 5 min and 3% was released from the larger particle size MSNP-Rif 100 in PBS at a pH of 7.4. It could be due to the larger surface areas of the smaller particle size and the attachment of RFC on the surface of the mesopores. Also, RFC was released at a similar rate from both MSNP-Rif 40 and MSNP-Rif 100 in acetate buffer at a pH of 5, which could be due to the poor solubility of RFC in more acidic conditions compared with pH of 7.4 [201].

Joyce *et al*. synthesized Hiroshima type MSNs using a sol–gel method for the encapsulation of RFC via centrifugation. The surface was modified with a hydrophobic component, hexane. The organic solvent and surfactant extraction process (calcination or solvent extraction) were optimized to produce various particle sizes and hydrophilicity. It was observed that the loading capacities of RFC increased with increasing hydrophobicity and particle size and therefore assumed that the drug loading capacity of RFC was dependent on the surface chemistry and particle size of the MSNs rather than the pore volume [202].

The effect of drug loading techniques, rotary evaporation and impregnation method on drug loading capacities of INZ, pyrazinamide, pyrazinoic acid and ethambutol (scheme 5) was also studied by Shawky *et al*. In their report, the rotary evaporation technique yielded a higher drug loading capacity than impregnation method. Also, the rotary evaporation method was independent of the nature of the drug, solvent used and MSN charge [203].

Nanoparticle-mediated delivery of anti-tubercular drugs provides distinct advantages over free drug molecules, like prolonged circulation and enhanced access of the therapeutic payload to the Mycobacterial tuberculosis-infected cells and tissues, consequently increasing the efficacy of the therapy. In addition, prolonged release of the drugs from nanosized carriers ensures persistent therapeutic concentrations of the drug for a more extended period, along with a better pharmacokinetic profile ensuring a less frequent dosage regimen and lower dose requirement [204].

Presently, various strategies have been employed for the successful and site-specific delivery of drugs to treat tuberculosis. Different ligands can be anchored to nanoparticles to carry numerous potential drugs for site-specific delivery. Lung targeting with non-ligand anchored nanoparticles offers potential challenges. The rapid exhalation of small-sized inhalable nanoparticles and mucociliary clearance of extra size inhalable nanoparticles create significant barriers for pulmonary targeting. Therefore, the ligand anchored nanomedicine delivery systems for effective anti-tubercular drug delivery to lungs and its internalization with a reduced reticuloendothelial system (RES) uptake [205].

Recently, INZ-loaded mannose-functionalized solid lipid nanoparticle (ISN-MAN SLNs) reinforced with sterylamine (SA) was designed and developed for effective alveolar macrophage targeting. The current study suggested that after *in vitro* cytotoxicity study in NCIH441 and dTHP-1 cell lines for both functionalized and unfunctionalized SLNs exhibits devoid of toxicity. Additionally, *in vitro* cellular uptake study revealed greater macrophagic internalization of mannosylated SLNs. Still, mannose treated pre-incubated cells exhibit a significant reduction of cellular uptake, evidencing receptors-dependent internalization of mannosylated SLNs [206].

The bid to improve anti-tubercular agents' drug loading and release performance led to the synthesis of Pretomanid and MCC7433, a novel nitroimidazopyrazinone analogue, and promising anti-tubercular agents that belong to the bicyclic nitroimidazole family. They suffer from poor aqueous solubility despite high cell permeability and require specialized formulations to be orally bioavailable. To address this limitation, we investigated the use of MSNs (MCM-41) as drug carriers. MCM-41 nanoparticles were synthesized using a sol–gel method, and the surface was modified with amine and phosphonate groups. The compounds were incorporated into the nanoparticles with rotary evaporation, leading to a high encapsulation efficiency of greater than or equal to 86% with approximately 10% loading (w/w) [199].

### 2.9. Drug loading and release methods

The efficiency of drug delivery systems is majorly determined by their loading capacities and release profiles [207]. Factors influencing mesoporous materials’ drug loading and release kinetics include pore size, surface area and surface functionalization [63]. The well-ordered structures of MSNs, high pore volume and large surface areas allow a high drug payload to be achieved [19]. Also, they possess efficient drug release mechanisms through surface modification. The silica framework and the nanocarriers' outermost surface can be used to attach surface functional groups that perform various roles in the interaction between drugs and nanocarriers, including the interaction between nanocarriers and the physiological environment [208]. Also, loaded drug molecules are protected effectively from physiological conditions and external environmental factors such as denaturation and enzymatic degradation due to the inorganic nature of MSNs and their non-swellable silica network [209].

#### 2.9.1. Drug loading methods

**Figure 6.**
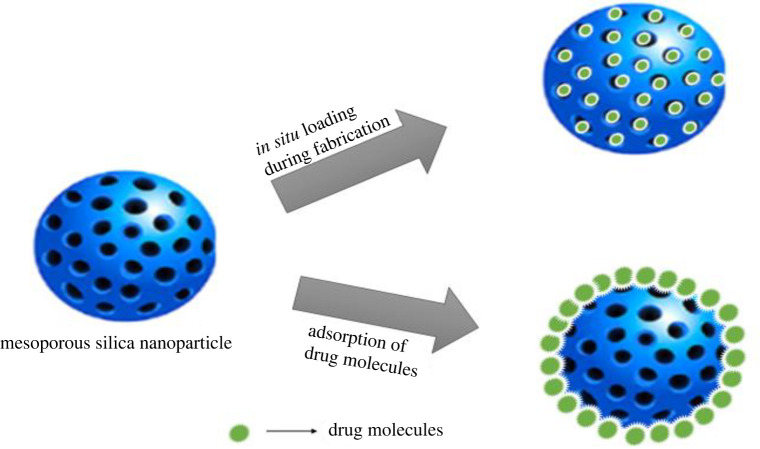
Diagram illustrating the *in situ* loading during fabrication and adsorption of drug molecules technique.

MSNs can maintain their structural integrity in organic solvents, making them suitable for encapsulating poor water-soluble drugs in a non-aqueous medium. They can also improve the dissolution rate of drugs [60]. Techniques used for loading drug molecules can be categorized into various classes. One of them is *in situ* loading during fabrication and adsorption of drug molecules within mesopores through chemisorption or physisorption, which is majorly influenced by the surface chemistry of the MSNs [207]. The physical adsorption method involves soaking MSNs in the desired drug solution with the aid of intermolecular interactions such as ionic, hydrophobic and dipole–dipole interactions, which is the most common and suitable approach for loading poor water-soluble drugs [12,210]. Figure 6 represents a schematic diagram illustrating the *in situ* loading during the fabrication and adsorption of drug molecules.

Other drug loading techniques are solvent-free and solvent-based methods [25]. Solvent-free methods include microwave irradiation [211], co-milling [212] and melting methods [213,214]. Solvent-based methods include incipient wetness impregnation [215,216], adsorptions [217,218], solvent evaporation [39,138,219–221], diffusions-supported loading [222], supercritical fluid technology [223], one-pot drug loading and synthesis procedure [224] and covalent grafting [225,226]. The drug entrapment/encapsulation efficiency (%EE) and drug loading content (%LC) of MSNs can be determined using the equations according to Dong *et al*. [227,228].

#### 2.9.2. Drug release methods

Drugs are released from their matrices through diffusion, erosion and desorption [229]. Their release mechanism depends on pore connectivity and size, the chemical composition of nanoparticle surfaces, physico-chemical properties and loading methods of the drugs. Their release kinetics from their matrices can be regulated by modifying their pore size and geometry, surface functionalization and drug loading methods [135]. Various methods are employed to study the *in vitro* release of drugs from nanoparticles, including dialysis bag diffusion, reverse dialysis bag diffusion [230], agitations followed by ultracentrifugation/centrifugation [231] and ultra-filtration [232,233].

### 2.10. Outlook for functionalized nanomesoporous silica materials as promising sustainable drug delivery modules for anti-tubercular agents

The treatment and management of tuberculosis, one of the world's most deadly infectious diseases, is faced with therapeutic challenges associated with conventional drug delivery systems. The main challenges include costly and prolonged treatment regimes, poor patient compliance, systemic toxicity and the use of poorly water-soluble drugs. Nanocarriers such as MSNs have provided a platform for overcoming these challenges. MSNs have significant merits such as controllable physico-chemical properties, low cytotoxicity and suitable biocompatibility, easy functionalization and high drug loading capacity. These properties can be used to enhance the therapeutic efficiency of anti-tubercular drugs, reduce their dosing frequency, minimize drug-related toxicity and achieve a better level of patient compliance. Their ability to enhance the solubility of poorly water-soluble drugs by controlling their physico-chemical properties and straightforward surface functionalization techniques have made them promising nanocarriers for enhancing the therapeutic efficiency of these drugs.

## Data Availability

No new data are associated with this article.
